# Development and Validation of a Scale to Measure Intimate Partner Violence Among Transgender and Gender Diverse Populations: Protocol for a Linear Three-Phase Study (Project Empower)

**DOI:** 10.2196/23819

**Published:** 2020-11-26

**Authors:** Rob Stephenson, Kieran Todd, Kristi E Gamarel, Erin E Bonar, Sarah Peitzmeier

**Affiliations:** 1 Center for Sexuality and Health Disparities and The School of Nursing University of Michigan Ann Arbor, MI United States; 2 Center for Sexuality and Health Disparities University of Michigan Ann Arbor, MI United States; 3 Center for Sexuality and Health Disparities and The School of Public Health University of Michigan Ann Arbor, MI United States; 4 Addiction Center Department of Psychiatry University of Michigan Ann Arbor, MI United States; 5 The Injury Prevention Center University of Michigan Ann Arbor, MI United States

**Keywords:** intimate partner violence, transgender, scale

## Abstract

**Background:**

Intimate partner violence (IPV) is approximately twice as prevalent among transgender and gender diverse individuals (those whose current gender identity does not match their sex assigned at birth) than among cisgender individuals (those whose gender aligns with their sex assigned at birth). However, most existing scales measuring IPV are not validated among transgender and gender diverse populations and do not consider the unique forms of IPV experienced by transgender and gender diverse individuals.

**Objective:**

This paper describes the protocol for Project Empower, a study that seeks to develop and validate a new scale to measure IPV as experienced by transgender and gender diverse adults. A new scale is necessary to improve the accuracy of IPV measurement among transgender and gender diverse populations and may inform the current tools used to screen and link to services for transgender and gender diverse people who experience or perpetrate IPV.

**Methods:**

The proposed new scale will be developed by a linear three-phase process. In Phase I, we will recruit a maximum of 110 transgender and gender diverse participants to participate in in-depth interviews and focus groups. Phase I will collect qualitative data on the experiences of IPV among transgender and gender individuals. After generating scale items from the qualitative data in Phase I, Phase II will conduct up to 10 cognitive interviews to examine understanding of scale items and refine wording. Phase III will then conduct a survey with an online recruited sample of 700 transgender and gender diverse individuals to validate the scale using factor analysis and examine the prevalence, antecedents, and linked health outcomes of IPV. This study will generate the first comprehensive IPV scale including trans-specific IPV tactics that has undergone robust mixed-methods validation for use in transgender and gender diverse populations, regardless of sex assigned at birth.

**Results:**

Project Empower launched in August 2019, with Phases I and II expected to be complete by late 2020. Phase III (survey of 700 transgender individuals) is expected to be launched in January 2021.

**Conclusions:**

A scale that more accurately captures the forms of IPV experienced by transgender and gender diverse people not only has the potential to lead to more accurate measurements of prevalence but also can identify unique forms of violence that may form the basis of IPV prevention interventions. Additionally, identifying the forms of IPV experienced by transgender and gender diverse people has the potential to lead to the refinement of clinical screening tools used to identify and refer those who experience and perpetrate violence in clinical settings.

**International Registered Report Identifier (IRRID):**

DERR1-10.2196/23819

## Introduction

### Background

Transgender and gender diverse individuals (individuals who identify as a gender different than the sex assigned to them at birth) are at 2.2 times the risk of physical intimate partner violence (IPV) and 2.5 times the risk of sexual IPV as compared with cisgender individuals [1]. Of the 27,715 transgender adults sampled in the 2016 US Transgender Survey (USTS) [2], 54% reported some form of lifetime IPV (35% reported physical IPV, 24% reported severe physical IPV, and 16% reported sexual IPV in their lifetime), and all rates were comparable to or greater than those documented in the general US population [2,3]. The few existing studies of IPV in transgender and gender diverse populations have shown links between IPV and a range of negative health outcomes, including posttraumatic stress disorder (PTSD) [4], avoidant coping behaviors, depressive symptoms [5,6], and HIV/sexually transmitted infection transmission risk behaviors, including transactional sex [7].

The experience or perpetration of IPV is commonly measured through scales that include lists of actions considered to constitute violence (ie, physical acts such as kicking or punching, sexual acts such as forced sex, and emotional acts such as verbal insults). However, many of the scales commonly used to measure IPV, for example, the Revised Conflicts Tactics Scale (CTS), were developed and validated with heterosexual cisgender populations and sometimes even only cisgender women [8]. Recent evidence demonstrates that there are acts of violence that are specific to the IPV experiences of transgender and gender diverse individuals, yet these acts are absent from commonly used IPV scales developed in cisgender populations. Omission of these transgender-specific acts from scales is problematic when measuring IPV in transgender and gender diverse populations, as these acts are common hallmarks of abuse for transgender and gender diverse individuals. In the 2016 USTS, 27% of participants reported experiencing some form of transgender-specific IPV in their lifetime, including their partner preventing them from accessing hormones (3%), telling them that they were not a “real” woman or man (25%), and threatening to “out” them as a transgender as a form of blackmail (11%) [2]. Perpetrators may undermine their partner’s gender identity and expression by intentionally misgendering them or hiding/damaging items (eg, chest binders, wigs, makeup, clothing, and prosthetics) [9]. These acts can lower self-esteem and confidence, rendering transgender and gender diverse individuals more vulnerable to abuse and less confident to go out in public and increasing their sense of isolation and dependency [1,10]. Existing IPV measures do not screen for these trans-specific abuse tactics; thus, they are likely insufficiently sensitive as IPV screening tools in transgender and gender diverse populations. Accurate measurements of IPV are essential for effective intervention development and evaluation of the impacts of interventions on behavioral change.

Several recent attempts have been made to create measurement tools that more accurately reflect IPV as experienced by transgender individuals. While not a transgender-specific scale, Woulfe and Goodman developed a seven-item scale of “identity abuse” with lesbian, gay, bisexual, transgender, and queer (LGBTQ)-specific items, such as “The person told me I deserve what I get because of my sexual orientation or gender identity” (8.2%) and “The person questioned whether my sexual orientation or gender identity was real” (28.3%) [11]. Transgender participants were more likely (49.3%) than sexual minority cisgender women (42.8%) or men (28.4%) to experience identity abuse in adulthood. Peitzmeier et al created the first transgender-specific IPV (T-IPV) scale and piloted it in a sample of 150 transmasculine individuals (ie, individuals assigned a female sex at birth who identify their gender on a spectrum of masculinity) [12]. The scale was then expanded to 10 potential items and tested again in two independent samples of transfeminine adults (ie, assigned a male sex at birth and identify with femininity), with factor analyses yielding an eight-item unidimensional scale with moderate to good fit [13]. Scale content represented a variety of domains of trans-specific abuse, including partner sabotaging gender transition (10% lifetime report), policing gender expression (21%), and emphasizing the undesirability of transpartners (22%). Additionally, Dyar et al [14] used data from a sample of 352 sexual and gender minority individuals assigned female at birth (SGM-AFAB) to adapt versions of the Conflict Tactics Scale–Revised, a measure of coercive control, and to test the newly developed SGM-Specific IPV Tactics Measure, with results providing initial evidence of the reliability and validity of each measure. This five-item method was designed for use with LGBTQ populations broadly and focused on outing and social isolation as domains. While these recent studies have attempted to create transgender- or LGBTQ-specific measurements of IPV, they are not without limitations. Items from these scales were developed through expert and community consultation and review of existing qualitative literature, but none were grounded in an in-depth qualitative study whose specific purpose was to elicit acts of IPV specific to transgender and gender diverse individuals by transgender and gender diverse survivors themselves, which may have restricted the content validity of the scale or impacted how the items were worded. These studies also relied solely on psychometric validation, usually in a restricted sample of either transfeminine or transmasculine individuals but not both, and did not include cognitive interviewing of the proposed scale items and other validation methods to ensure that they accurately captured the experiences of transgender individuals.

This study seeks to fill this gap through the development and validation of a scale that comprehensively accounts for both forms of IPV that may be experienced by individuals of all gender identities (ie, forced sex) and forms of IPV that are unique to transgender and gender diverse individuals. This paper describes the protocol for *Project Empower*, a project to develop and validate a new scale to measure IPV as experienced by transgender and gender diverse populations aged 15 years and above in the United States. A new scale is necessary to improve the accuracy of IPV measurement among transgender and gender diverse populations, and may inform the current tools used to screen and link to services for transgender and gender diverse adults who experience or perpetrate IPV. This scale will be grounded in *de novo* qualitative data collection specifically designed to elicit the types of abuse experienced by transgender and gender diverse survivors of IPV, and be comprehensively validated through qualitative focus groups, cognitive interviews, and finally quantitative psychometric validation.

### Theoretical Framework for Scale Development

**Figure 1:**
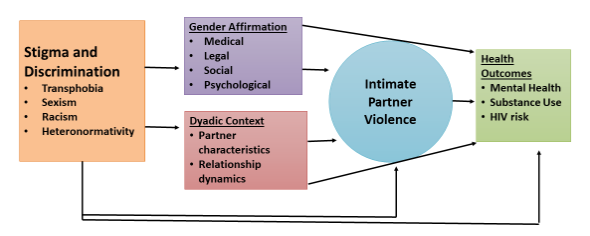
Conceptual model of intimate partner violence among transindividuals.

The conceptual model of IPV among transindividuals is presented in Figure 1. Our conceptual framework is guided by adaptions of the gender minority stress model and gender affirmation framework. According to the gender minority stress model, individuals who do not conform to societal norms regarding gender roles, expression, and identities are vulnerable to discrimination and stigma, which can affect emotions, cognitions, and health behaviors [15]. In accordance with the gender affirmation framework, gender minority stressors may increase the need for gender affirmation (eg, feeling safe, recognized, and supported in gender identity and expression [16,17]), particularly in intimate relationships. Gamarel et al developed and validated a measure of relationship stigma with transgender women, which measures enacted stigma and anticipated stigma experienced by transgender women in their intimate relationships [18]. Transgender-specific discrimination and relationship stigma have been associated with reduced relationship quality, as well as increased substance use behaviors and psychological distress for transgender women and their cisgender male partners [18,19]. Thus, IPV may be a result of societal oppression, transgender-specific discrimination, and relationship stigma, whereby partners of transgender individuals may withhold gender affirmation as a power and control tactic. The conceptual framework for our study draws upon these frameworks and hypothesizes IPV to be a function of multiple interlocking forms of stigma and discrimination (ie, antitransgender stigma), gender affirmation, and dyadic and individual factors. We hypothesize that the presence of any of these factors may influence IPV in one of the following two ways: (1) the creation of stress leading to an increased propensity for violence and (2) lowered access to and greater need for gender affirmation from a partner leading to greater vulnerability to or acceptance of violence. The model will be used to guide the collection of qualitative and quantitative data to understand the unique forms and antecedents of IPV among transgender and gender diverse individuals.

## Methods

### Trial Registration, Ethics, Consent, and Institutional Board Approval

This study has been reviewed and approved by the University of Michigan Institutional Review Board (IRB# HUM00171509). A certificate of confidentiality has been obtained from the National Institute of Child Health and Human Development, and a waiver of parental consent/assent will be obtained for participants who are 15 to 17 years old.

### Study Design

#### Overview of Study Design

The proposed new scale will be developed by a linear three-phase process. Phase I consists of collecting qualitative data (ie, in-depth interviews and focus group discussions) with up to 110 transgender and gender diverse individuals aged over 18 years (for the in-depth interviews) and aged 15 and above (for the focus group discussions) to better understand the experiences of IPV, and it will provide content items for the proposed new scale. After extracting scale items from the qualitative data in Phase I, Phase II will consist of a maximum of 10 cognitive interviews to examine construct and content validity of scale items. Phase III will be a survey with an anonymous online sample of 700 transgender and gender diverse individuals aged 15 years and above to examine the prevalence, antecedents, and linked health outcomes of IPV and psychometrically validate a comprehensive IPV scale validated for use in transgender and gender diverse populations. Participation in the in-depth interviews is restricted to participants over the age of 18 years, as the interviews potentially involve disclosures of personal experiences of violence, and restricting participation to adults prevents the disclosure of child/minor abuse. Phase I and II data collection began in March 2020 and has continued throughout the COVID-19 pandemic that began in the United States in March 2020. The study has not experienced problems in recruiting participants for either Phase I or II. To recognize the potential for the additional stress of the pandemic to create contexts or triggers for IPV, we will add the measure of COVID-related stressors (ie, employment loss) to the Phase III survey.

#### Participants

For all phases, participants must be (1) transgender or gender diverse (ie, defined as a difference in the sex assigned at birth and current gender identity), (2) aged 15 years and above, (3) currently residing in the United States, (4) having access to a computer, tablet, or smartphone, (5) having at least one intimate partner in the past 5 years, and (6) able to participate in the study in English. For the in-depth interviews, additional eligibility criteria are having experienced IPV in the previous 5 years, using the IPV screener from the USTS, and being over 18 years of age [2].

#### Recruitment

The same recruitment process will be used for Phases I to III. We will recruit participants using racially and ethnically diverse banner advertising on mobile dating apps (eg, Fet-Life) and social networking websites (eg, Facebook and Instagram). Online advertising will be supplemented with placing advertisements on the webpages of leading transgender health and rights websites, tweeting links to study information with transgender-specific hashtags, and working with transgender activists and organizations to tweet and promote the study on their social media profiles. Recruitment will be limited to those residing in the United States. Individuals who click on the banner advertisements will be taken to the study website for more information. Interested individuals will complete an online consent form and eligibility screener. Eligible participants will then enroll by providing their name and email. Participants who do not consent, do not meet the eligibility criteria, or do not provide valid contact information will be excluded from the study.

#### Protection of Human Subjects

Throughout the phases of the study, we will take several important steps to ensure the safety and privacy of the participants. The synchronous focus group discussions, in-depth interviews, and cognitive interviews will be conducted using Health Insurance Portability and Accountability Act (HIPAA)-compliant video-based teleconferencing software (Zoom). Focus group discussions carry the risk of disclosure of personal information by other study participants. Participants will be informed that no personal information should be shared and will be asked to respect the confidentiality of other participants and not repeat discussions outside of the online chat room. They will also be instructed to sit in a private place when participating in the focus group discussion so that other people are not able to see images, hear audio, or view text contributed by the other participants in the focus group discussion. All study staff will follow a robust set of safety procedures to make sure that participants receive a high level of monitoring. Because there is potential for psychological discomfort due to the research topic, participants will be informed that they may refuse to answer any question and that they may end their participation at any point, without penalty. Participants experiencing distress during the interviews will be offered brief breaks and survey participants will be reminded that they can take breaks during the self-administered online survey. In the unlikely event that a participant experiences considerable distress (ie, discloses suicidal ideation or self-harm attempts), research staff are trained to assess risk for self-harm and suicidality, with guidance from an on-call licensed clinician and the study Principal Investigators.

Given the remote nature of participation, participants who are at acute risk or are interested in connecting to support services will be connected with a national crisis line which can facilitate local referral as needed. If any person is judged by project investigators to be at acute risk of danger to self or others, or judged to be in grave danger due to medical or other conditions, the decision will be made to break confidentiality in order to inform authorities to intervene in preventing an adverse event. During the Phase III survey, participants will self-administer surveys. Those participants who report that they are experiencing indicators of poor mental health (ie, emotional distress or depressive symptomology) will be shown a page of resources at the end of the survey and will be asked to acknowledge that they have received and read the resources.

#### Phase I Study Procedures

The first phase of the project will include 20 to 30 semistructured in-depth interviews and eight focus group discussions. All qualitative data collection will involve a diverse sample of transfeminine, transmasculine, and nonbinary individuals. We will use purposive sampling to increase the racial and sexual orientation diversity of the participants [20]. Upon initial saturation, theoretical sampling and additional interviews (up to 30) may be conducted to expand upon or confirm findings from initial interviews if necessary [21]. In order to account for different types of data, we will use a two-pronged approach for conducting the focus group discussions. There will be asynchronous and synchronous focus group designs, with the aim of conducting four of each type of focus group discussion. The in-depth interview and four synchronous focus group discussions will be conducted using HIPAA-compliant video-based teleconferencing software. The four asynchronous focus group discussions will be conducted via FocusGroupIt for text-based focus group discussions, a format that works well for maximal anonymity on sensitive topics, and to allow participation from geographically dispersed participants across multiple time zones. Each focus group discussion will contain between 5 and 10 participants. Synchronous focus group discussions and in-depth interviews will be conducted virtually, using the HIPAA-compliant secure Zoom platform. After screening and enrolling, study staff will call participants to schedule the focus group discussion or in-depth interview.

The in-depth interview and focus group discussion will serve unique purposes. The in-depth interview will focus on collecting IPV narratives from survivors of IPV and understanding the specific abusive behaviors that took place, the perceived triggers of violent events, and how violence behaviors evolved over the course of the relationship and over the course of the individual’s gender transition. After the in-depth interviews are transcribed, we will extract behaviors considered abusive by survivor participants and craft potential screening items to screen for these behaviors, using in vivo language as provided by survivors to the extent possible. The focus group discussion will enroll both individuals who have and who have not experienced IPV, and explore group norms and perceptions of IPV. The moderator will ask the participants if each of the potential screening items identified in the in-depth interview is considered violent, and will ask participants to name other actions that may be considered violent in a relationship, probing around actions specific to the transgender experience. We will also ask participants about items from commonly used scales, such as the CTS, and from other trans-specific IPV scales that have been developed in the past [2,13,14]. We will then ask focus group discussion participants to talk about what other behaviors they would consider abuse that we have not yet asked about. This process will ensure that the full range of IPV as experienced by transgender and gender diverse individuals will be captured by the scale based on qualitative data collection capturing the lived experiences of transgender and gender diverse participants. The qualitative data collection will be conducted by a transgender moderator. Participants will receive US $50 compensation sent via a Mastercard gift card for participation in the in-depth interview and US $30 for participation in the focus group. Participants who participate in the in-depth interview will not be eligible for the focus group discussion.

#### Phase II Study Procedures

The first task in Phase II will be to finalize the items for the new scale to measure IPV among transgender individuals. Focus group discussion and in-depth interview transcripts will be reviewed by three researchers in order to ensure that all unique forms of IPV mentioned by the participants were extracted to inform survey instrument development. The number of unique IPV items generated is expected to be several dozen. After generating the survey items, a maximum of 10 cognitive interviews will be conducted to explore item comprehension of the potential scale items. The aim of the cognitive interviews is to refine the language used in each of the scale items and ensure that participants understand the items in the manner intended. Participants for the cognitive interviews will be recruited in the same manner as participants in Phase I. Cognitive interviews will be conducted virtually, using the HIPAA-compliant Zoom platform. Participants will be asked to read the scale items and then repeat the meaning back in their own words. If there is a discrepancy in meaning, the interviewer will explain the intended meaning to the participant and ask the participant to rephrase the scale item to achieve the desired meaning [22]. We will also ask participants to “talk aloud” their thought process in how they respond to the questions and test assumptions inherent to the scale, such as understanding of an “intimate partner.” Cognitive interview participants will receive US $30. Participants in the in-depth interview and focus group discussion will not be eligible to participate in the cognitive interviews.

#### Phase III

##### Phase III Study Procedures

After completing this process of generating potential IPV scale items and other variables of interest grounded in the Phase I qualitative data, we will conduct an online survey of 700 transgender and gender diverse individuals. Eligibility criteria, recruitment, and enrollment strategies are the same as those in Phases I and II, and the survey will sample those aged 15 years and above. Once eligible and enrolled, participants will be emailed a link to a secure survey. The survey can only be taken once to prevent duplicates. Participants will receive US $40.

##### Sample Size for Phase III

We will enroll 700 transgender individuals aged 15 years and above. Previous studies of IPV among transgender individuals have shown the prevalence of physical IPV to be in the range of 18% to 47%. Each of these studies has relied upon measures of IPV that are not transgender-specific, and we expect our prevalence estimates to be higher. A sample size of 700 is sufficient to detect a roughly 20% difference in IPV prevalence (ie, odds ratio 1.5-1.9) between two subgroups in our sample (eg, transfeminine, transmasculine, and nonbinary) with >80% power.

#### Survey Instruments

##### IPV

With regard to *experience and perpetration of IPV and scale development*, participants will view a list of acts that may constitute IPV and respond to the question, “Which of the following items would you consider to be violent or abusive if a sexual/romantic partner perpetrated them against you without your consent?” For each behavior, they will report if they have experienced or perpetrated it in the past 12 months and lifetime, and will report the number of times they have experienced each item in the past 12 months. With regard to *partnership-level experience of IPV*, for each intimate partner reported in the past 3 years (up to three partners), we will assess IPV victimization and perpetration. For partnerships in which victimization or perpetration is reported, respondents will complete the Controlling Behaviors Scale to assess IPV typology (eg, intimate terrorism versus common couples’ violence [23]) and the injury scale of the CTS2 to measure the severity of physical violence [8]. With regard to *help seeking*, participants who experienced IPV will be asked in which ways they sought help or assistance from a variety of sources, including friends, family, physicians/medical workers, counselors/psychiatrists, social workers, and law enforcement.

##### Stigma and Discrimination

Gender minority specific stigma will be assessed using validated subscales from the Gender Minority Stress and Resilience Scale (GMSRS) [24]. Sexual minority stigma will be assessed with the Intersectional Discrimination Index [25] and the Everyday Experiences of Discrimination (Sexual Orientation) Scale [26-28]. Racial/ethnic stigma will be captured with the Brief Perceived Ethnic Discrimination Questionnaire (Community Version) [29], the Everyday Experiences of Discrimination (Race/Ethnicity) Scale [26-28], and the Group Membership Questionnaire [30]. Experiences of sexism will be assessed with the Daily Sexist Events Scale [31] and the Internalized Misogyny Scale [32,33].

##### Gender Affirmation

Participants will also complete a gender affirmation scale that assesses domains, including need for, access to, and satisfaction with legal affirmation (eg, name changes), medical affirmation (eg, hormones and surgery), social affirmation (ie, correct pronouns), and psychological affirmation (eg, level of femininity/masculinity) [34]. We will also assess satisfaction with gender transition and gender comfort [17,35].

##### Dyadic Context

Participants will be asked to list their most recent intimate partners (defined as someone the participant felt emotionally, romantically, or sexually close to) in the past 3 years (up to three partners). Participants will be asked to provide nicknames or initials for their partners, as opposed to identifying information. For each partner, we will ask the gender, age, race, sexual orientation, education, and outness. Participants will complete measures of relationship functioning, which include the Dyadic Adjustment scale [18,36] Commitment scale [37], and Power and Attitudes in Relationships scale [38]. We will assess stigma experienced at the dyadic level with a validated measure of relationship stigma [18].

##### Demographics

Age, education, race, ethnicity, sexual orientation, employment, health insurance, health care access, and state of residence will be measured. Both sex assigned at birth and current gender identity will be collected. Current gender identity will include options for male, female, transmasculine, and transfeminine, as well as categories for genderqueer/gender nonconforming, nonbinary, agender/gender fluid, and participant-driven response.

##### Health Outcomes

The AIDS Risk-Behavior Assessment (ARBA) adapted to be relevant to transgender bodies and relationships [39] will be used to collect information on sexual behaviors with the three most recent partners in the past 3 years to enable partner-by-partner analyses. Participants will also be asked if they felt able to negotiate for condom use and/or contraceptive use with each partner. In addition to partner-level information on sexual behavior, participants will also be asked about their sexual behavior in the past 12 months with any partner. *History of HIV testing* will include measures of frequency, method of testing, and linkage to care (if HIV positive). *Substance use* measures will assess the use and frequency of use of alcohol and other drugs in the past 3 months, and are based on prior work [40-43]. We will measure nonprescribed and prescribed *hormone use*, age of starting hormones, types of hormones, dosage/regimen, and adherence to prescribed hormones. We will use the Brief Symptom Inventory [44] to measure current depressive symptomology and anxious symptomology. PTSD symptoms will be assessed with a scale [45,46] used in studies with transgender communities [4]. *Nonsuicidal self-injury* will be measured with the Inventory Statements about Self-Injury (ISAS) [47]. *General physical health* will be measured using the Patient-Reported Outcomes Measurement Information System (PROMIS) measure [47].

##### COVID-19 Stressors

To recognize the potential for the COVID-19 pandemic to create additional stress, the survey will assess the following COVID-19-related stressors: loss of employment or income, loss or changes in housing, changes in access to health care, increased participation in child or elder care, participation in social distancing, and feelings of anxiety, loneliness, and isolation specifically linked to COVID-19.

#### Phase III Data Analysis

We will reduce scale items by dropping items not considered to be violent by the majority (>60%) of respondents. To validate the scale and identify factors/subscales, we will conduct exploratory factor analysis (EFA) using victimization data for the past 12 months [48] from a randomly selected sample of half of the participants. We will not a priori propose a factor structure for the scale, but we will compare the resultant factor structure with those of commonly used IPV scales. We will examine whether the data are suitable for factor analysis with the Kaiser-Meyer-Olkin test and Bartlett test of sphericity [49,50]. Factor retention will be decided by examining eigenvalues, scree plots, and interpretability of factors [49]. Oblique and orthogonal rotations will be examined to determine the best solution. The reliability of each factor/subscale will be assessed by calculating Cronbach alpha, with adequate reliability indicated if Cronbach alpha is >.70 in the overall sample and among subgroups (ie, transmasculine, transfeminine, and nonbinary participants) [51]. Items may be reduced based on statistical (items should increase subscale alpha, have high item-total correlations, and load highly onto a single factor), theoretical (maintaining items for content validity), and practical (scale length) criteria [8,48,52]. As an additional exploratory analysis, we will conduct EFA separately for different subgroups of respondents (eg, transmasculine and transfeminine) to identify potential variations in scale content. We will compare the factor structures of the EFA conducted for subgroups of the sample, although the intent remains to create a single scale that represents the IPV experiences of all transgender and gender diverse individuals. We will then conduct a confirmatory factor analysis of the retained items with the participants not in the EFA sample. Model fit will be evaluated by looking for a root mean square error of approximation <0.06, confirmatory factor index >0.90, and standardized root mean squared error close to 0.08 [53,54]. Convergent validity will be assessed by measuring the correlation with existing IPV measures, use of domestic violence services, and relationship functioning. Analyses will produce a validated IPV scale with subscales corresponding to different forms (eg, physical, sexual, etc) that comprehensively measure IPV in transgender populations.

##### Quantifying Individual-Level Factors Associated With IPV Experience and Perpetration

We will define the following outcomes for the past 12 months and lifetime referent periods: (1) experiencing any form of IPV, (2) experiencing each domain of IPV identified during scale development (eg, physical IPV and sexual IPV), (3) perpetrating any form of IPV, and (4) perpetrating each domain of IPV. Key covariates will be stigma and discrimination, gender affirmation, and demographic variables. Bivariate associations between each of the outcomes and covariates will be examined. The goal of this stage of the analysis will be to identify the prevalence of forms of IPV in different subgroups, including differences in the IPV by different subgroups (eg, transmasculine, transfeminine, and/or nonbinary identity), to assess IPV disparities within transgender and gender diverse communities. Additionally, multivariate logistic regression models will be fit to determine the independent risk of IPV associated with each individual-level characteristic.

##### Quantifying Partner-Level and Dyadic Factors Associated With IPV Experience and Perpetration

This analysis will use data reported for each recent partnership (up to three per participant). The outcomes will be the experience and perpetration of any IPV and of each form of IPV during each partnership. Covariates will include individual-level characteristics as above in the individual-level models, partner characteristics (eg, partner gender), and dyadic characteristics (eg, dyadic adjustment and relationship stigma). Multilevel logistic regression models with random effects will be fitted with partners clustered under individuals [55].

##### Quantifying Associations Between Experience and Perpetration of IPV and Health Outcomes

This analysis will use individual-level models to assess associations between IPV and health outcome measures enumerated above, including sexual health, mental health, substance use, and physical health outcomes. Logistic, multinomial, or linear models will be employed depending on the outcome. The key covariates in each model will be the experience or perpetration of IPV in the past 12 months and lifetime, adjusting for individual-level covariates. Then, partner-level multilevel models will be fit to quantify associations between IPV experience or perpetration within a given partnership and sexual behaviors for that partnership (eg, condom use).

## Results

Project Empower launched in September 2019, with Phases I and II expected to be complete by late 2020. Phase III (survey of 700 transgender individuals) is expected to be launched in January 2021.

## Discussion

Transgender and gender diverse people in the United States face adverse physical and mental health outcomes compared with cisgender populations [5,56-67]. There is a wealth of literature illustrating the epidemic rates of psychological distress [2], depression [68-74], and suicidal ideation [73,75-78], as well as poor self-rated physical health [2], high rates of HIV and other sexually transmitted infections [60,79-82], and elevated risk for chronic disease [83-85] among transgender and gender diverse individuals. These disparities may be driven in part by disproportionate rates of violence, including IPV, making sensitive and accurate screening, prevention, and response for IPV in transgender and gender diverse populations critical.

Central to the ability to develop efficacious interventions for the primary or secondary prevention of IPV is our ability to correctly define IPV as it is experienced by transgender and gender diverse individuals. Current commonly used IPV scales were developed primarily for use in heterosexual cisgender populations and do not necessarily capture the lived experiences of transgender and gender diverse individuals and the forms of IPV that they may uniquely experience. Prior work has developed and validated scales to measure IPV as experienced by gay and bisexual men [86] and developed and validated an IPV scale specifically for sexual and gender communities [14], and preliminary work has been performed to create an IPV scale specific to the unique experiences of transgender and gender diverse individuals [12]. Our current work extends this previous work by considering the experiences of transgender and gender diverse individuals as it relates to the experience of IPV. A scale that more accurately captures the forms of IPV experienced by transgender and gender diverse people not only has the potential to lead to more accurate measurements of prevalence, but also can identify unique forms of violence that may form the basis of violence prevention interventions. Additionally, identifying the forms of IPV experienced by transgender and gender diverse individuals has the potential to lead to the refinement of clinical screening tools that are used to identity and refer those who experience and perpetrate violence in clinical settings.

## Abbreviations

CTS: Conflicts Tactics Scale
EFA: exploratory factor analysis
HIPAA: Health Insurance Portability and Accountability Act
IPV: intimate partner violence
LGBTQ: lesbian, gay, bisexual, transgender, and queer
PTSD: posttraumatic stress disorder
SGM: sexual and gender minority
USTS: US Transgender Survey

## References

[1] Peitzmeier SM, Malik M, Kattari SK, Marrow E, Stephenson R, Agénor M, Reisner SL (2020). Intimate Partner Violence in Transgender Populations: Systematic Review and
Meta-analysis of Prevalence and Correlates. Am J Public Health.

[2] James S, Herman J, Rankin S, Keisling M, Mottet L, Anafi M (2016). The Report of the 2015 U.S. Transgender Survey.

[3] Black M, Basile K, Breiding M, Smith S, Walters M, Merrick M, Chen J, Stevens M (2011). The National Intimate Partner and Sexual Violence Survey (NISVS): 2010 Summary
Report.

[4] Reisner SL, White Hughto JM, Gamarel KE, Keuroghlian AS, Mizock L, Pachankis JE (2016). Discriminatory experiences associated with posttraumatic stress disorder
symptoms among transgender adults. J Couns Psychol.

[5] White Hughto JM, Pachankis JE, Willie TC, Reisner SL (2017). Victimization and depressive symptomology in transgender adults: The
mediating role of avoidant coping. J Couns Psychol.

[6] Goldenberg T, Jadwin-Cakmak L, Harper GW (2018). Intimate Partner Violence Among Transgender Youth: Associations with
Intrapersonal and Structural Factors. Violence Gend.

[7] Logie CH, Wang Y, Lacombe-Duncan A, Jones N, Ahmed U, Levermore K, Neil A, Ellis T, Bryan N, Marshall A, Newman PA (2017). Factors associated with sex work involvement among transgender women in
Jamaica: a cross-sectional study. Journal of the International AIDS Society.

[8] Straus MA, Hamby SL, Boney-McCoy S, Sugarman DB (2016). The Revised Conflict Tactics Scales (CTS2). Journal of Family Issues.

[9] Munson M, Cook-Daniels L (2003). Transgender/SOFFA: Domestic Violence/Sexual Assault Resource Sheet.

[10] Guadalupe-Diaz XL, Anthony AK (2016). Discrediting Identity Work: Understandings of Intimate Partner Violence by
Transgender Survivors. Deviant Behavior.

[11] Woulfe JM, Goodman LA (2018). Identity Abuse as a Tactic of Violence in LGBTQ Communities: Initial
Validation of the Identity Abuse Measure. J Interpers Violence.

[12] Peitzmeier SM, Hughto JM, Potter J, Deutsch MB, Reisner SL (2019). Development of a Novel Tool to Assess Intimate Partner Violence Against
Transgender Individuals. J Interpers Violence.

[13] Peitzmeier S, Reisner S, Cooney E, Humes E, Wirtz A (2019). Validation of a scale to measure transgender-specific psychological intimate
partner violence.

[14] Dyar C, Messinger AM, Newcomb ME, Byck GR, Dunlap P, Whitton SW (2019). Development and Initial Validation of Three Culturally Sensitive Measures
of Intimate Partner Violence for Sexual and Gender Minority Populations. J Interpers Violence.

[15] Hendricks M, Testa R (2012). A conceptual framework for clinical work with transgender and gender
nonconforming clients: An adaptation of the Minority Stress Model. Professional Psychology: Research and Practice.

[16] Sevelius JM (2013). Gender Affirmation: A Framework for Conceptualizing Risk Behavior among
Transgender Women of Color. Sex Roles.

[17] Glynn TR, Gamarel KE, Kahler CW, Iwamoto M, Operario D, Nemoto T (2016). The role of gender affirmation in psychological well-being among
transgender women. Psychol Sex Orientat Gend Divers.

[18] Gamarel KE, Reisner SL, Laurenceau J, Nemoto T, Operario D (2014). Gender minority stress, mental health, and relationship quality: a dyadic
investigation of transgender women and their cisgender male partners. J Fam Psychol.

[19] Reisner SL, Gamarel KE, Nemoto T, Operario D (2014). Dyadic effects of gender minority stressors in substance use behaviors
among transgender women and their non-transgender male partners. Psychol Sex Orientat Gend Divers.

[20] Patton MQ (1990). Qualitative Research & Evaluation Methods.

[21] Dworkin S (2012). Sample size policy for qualitative studies using in-depth
interviews. Arch Sex Behav.

[22] Beatty P, Willis G (2007). Research Synthesis: The Practice of Cognitive Interviewing. Public Opinion Quarterly.

[23] Graham-Kevan N, Archer J (2003). Intimate terrorism and common couple violence. A test of Johnson's
predictions in four British samples. J Interpers Violence.

[24] Testa R, Habarth J, Peta J, Balsam K, Bockting W (2015). Development of the Gender Minority Stress and Resilience
Measure. Psychology of Sexual Orientation and Gender Diversity.

[25] Scheim AI, Bauer GR (2019). The Intersectional Discrimination Index: Development and validation of
measures of self-reported enacted and anticipated discrimination for intercategorical
analysis. Soc Sci Med.

[26] Clark R, Coleman AP, Novak JD (2004). Brief report: Initial psychometric properties of the everyday
discrimination scale in black adolescents. J Adolesc.

[27] Forman TA, Williams DR, Jackson JS, Gardner C (1997). Race, place, and discrimination. Perspectives on Social Problems.

[28] Essed P (1991). Analyzing Accounts of Racism. Understanding Everyday Racism: An Interdisciplinary Theory.

[29] Brondolo E, Kelly KP, Coakley V, Gordon T, Thompson S, Levy E, Cassells A, Tobin JN, Sweeney M, Contrada RJ (2005). The Perceived Ethnic Discrimination Questionnaire: Development and
Preliminary Validation of a Community Version1. J Appl Social Pyschol.

[30] Contrada RJ, Ashmore RD, Gary ML, Coups E, Egeth JD, Sewell A, Ewell K, Goyal TM, Chasse V (2001). Measures of Ethnicity-Related Stress: Psychometric Properties, Ethnic Group
Differences, and Associations With Well-Being1. J Appl Social Pyschol.

[31] Swim J, Cohen L, Hyers L (1998). Experiencing everyday prejudice and discrimination. Prejudice: The Target's Perspective.

[32] Piggott M (2004). Double jeopardy: Lesbians and the legacy of multiple stigmatized
identities. Swinburne University.

[33] Szymanski DM, Dunn TL, Ikizler AS (2014). Multiple minority stressors and psychological distress among sexual
minority women: The roles of rumination and maladaptive coping. Psychology of Sexual Orientation and Gender Diversity.

[34] Sevelius J, Chakravarty D, Neilands TB, Keatley J, Shade SB, Johnson MO, Rebchook G, HRSA SPNS Transgender Women of Color Study Group (2019). Evidence for the Model of Gender Affirmation: The Role of Gender
Affirmation and Healthcare Empowerment in Viral Suppression Among Transgender Women of
Color Living with HIV. AIDS Behav.

[35] Bockting WO, Miner MH, Swinburne Romine RE, Hamilton A, Coleman E (2013). Stigma, Mental Health, and Resilience in an Online Sample of the US
Transgender Population. Am J Public Health.

[36] Spanier GB (1976). Measuring Dyadic Adjustment: New Scales for Assessing the Quality of
Marriage and Similar Dyads. Journal of Marriage and the Family.

[37] Tzeng OC (1994). Family Relations. Measurement of Love and Intimate Relations: Theories, Scales, and Applications for
Love Development, Maintenance, and Dissolution.

[38] Sherman S, Gielen A, McDonnell K (2000). Brief Report: Power and Attitudes in Relationships (PAIR) Among a Sample of
Low-Income, African-American Women: Implications for HIV/AIDS Prevention. Sex Roles.

[39] Reisner S, Deutsch M, Cavanaugh T, Pardee D, White HJ, Peitzmeier S, McLean S, Mimiaga M, Panther L, Potter J (2016). Best practices for obtaining a sexual health history with trans masculine
individuals: Lessons learned from self-administered surveys and provider-collected
clinical interview data. Proceedings of the 24th World Professional Association for Transgender Health
Biennial Symposium: Growing Empowerment, Expertise, Evidence; WPATH'16.

[40] Saunders J, Aasland O, Babor T, de la Fuente JR, Grant M (1993). Development of the Alcohol Use Disorders Identification Test (AUDIT): WHO
Collaborative Project on Early Detection of Persons with Harmful Alcohol
Consumption--II. Addiction.

[41] NIDA Alcohol, Smoking, and Substance Involvement Screening Test: NM-ASSIST.

[42] WHO ASSIST Working Group (2002). The Alcohol, Smoking and Substance Involvement Screening Test (ASSIST):
development, reliability and feasibility. Addiction.

[43] Grant BF, Chu A, Sigman R, Amsbary M, Kali J, Sugawara Y, Jiao R, Ren W, Goldstein R (2003). National Epidemiologic Survey on Alcohol and Related Conditions-III (NESARC- III):
Source and Accuracy Statement.

[44] Derogatis LR, Melisaratos N (2009). The Brief Symptom Inventory: an introductory report. Psychol. Med.

[45] Ouimette P, Wade M, Prins A, Schohn M (2008). Identifying PTSD in primary care: comparison of the Primary Care-PTSD
screen (PC-PTSD) and the General Health Questionnaire-12 (GHQ). J Anxiety Disord.

[46] Primary Care PTSD Screen for DSM-5 (PC-PTSD-5). U.S. Department of Veterans Affairs.

[47] Cella D, Riley W, Stone A, Rothrock N, Reeve B, Yount S, Amtmann D, Bode R, Buysse D, Choi S, Cook K, Devellis R, DeWalt D, Fries JF, Gershon R, Hahn EA, Lai J, Pilkonis P, Revicki D, Rose M, Weinfurt K, Hays R, PROMIS Cooperative Group (2010). The Patient-Reported Outcomes Measurement Information System (PROMIS)
developed and tested its first wave of adult self-reported health outcome item banks:
2005-2008. J Clin Epidemiol.

[48] Hegarty K, Bush R, Sheehan M (2005). The Composite Abuse Scale: Further Development and Assessment of
Reliability and Validity of a Multidimensional Partner Abuse Measure in Clinical
Settings. Violence.

[49] Tabachnick B, Fidell L (2007). Using multivariate statistics.

[50] Cudeck R, Tinsley H, Brown S (2000). Exploratory factor analysis. Handbook of Applied Multivariate Statistics and Mathematical Modeling.

[51] Hinkin TR (2016). A Brief Tutorial on the Development of Measures for Use in Survey
Questionnaires. Organizational Research Methods.

[52] DeVellis R (2017). Scale development: Theory and applications.

[53] Yu CY, Muthen BO (2002). Evaluation of model fit indices for latent variable models with categorical
and continuous outcomes.

[54] Schumacker R, Lomax R (2004). A Beginner's Guide to Structural Equation Modeling.

[55] Snijders T, Bosker R (1999). Multilevel analysis: An introduction to basic and applied multilevel
analysis. Multilevel Analysis.

[56] Durwood L, McLaughlin KA, Olson KR (2017). Mental Health and Self-Worth in Socially Transitioned Transgender
Youth. J Am Acad Child Adolesc Psychiatry.

[57] Valentine SE, Peitzmeier SM, King DS, O'Cleirigh C, Marquez SM, Presley C, Potter J (2017). Disparities in Exposure to Intimate Partner Violence Among
Transgender/Gender Nonconforming and Sexual Minority Primary Care
Patients. LGBT Health.

[58] Seelman KL, Colón-Diaz MJ, LeCroix RH, Xavier-Brier M, Kattari L (2017). Transgender Noninclusive Healthcare and Delaying Care Because of Fear:
Connections to General Health and Mental Health Among Transgender Adults. Transgend Health.

[59] Bockting W, Coleman E, Deutsch MB, Guillamon A, Meyer I, Meyer W, Reisner S, Sevelius J, Ettner R (2016). Adult development and quality of life of transgender and gender
nonconforming people. Current Opinion in Endocrinology & Diabetes and Obesity.

[60] (2017). HIV and Transgender People. Centers for Disease Control and Prevention.

[61] Gamarel KE, Reisner SL, Laurenceau J, Nemoto T, Operario D (2014). Gender minority stress, mental health, and relationship quality: a dyadic
investigation of transgender women and their cisgender male partners. J Fam Psychol.

[62] Meyer IH, Brown TN, Herman JL, Reisner SL, Bockting WO (2017). Demographic Characteristics and Health Status of Transgender Adults in
Select US Regions: Behavioral Risk Factor Surveillance System, 2014. Am J Public Health.

[63] Reisner SL, Gamarel KE, Dunham E, Hopwood R, Hwahng S (2013). Female-to-male transmasculine adult health: a mixed-methods community-based
needs assessment. J Am Psychiatr Nurses Assoc.

[64] Reisner SL, Greytak EA, Parsons JT, Ybarra ML (2015). Gender minority social stress in adolescence: disparities in adolescent
bullying and substance use by gender identity. J Sex Res.

[65] Reisner SL, Katz-Wise SL, Gordon AR, Corliss HL, Austin SB (2016). Social Epidemiology of Depression and Anxiety by Gender
Identity. J Adolesc Health.

[66] Stephenson R, Riley E, Rogers E, Suarez N, Metheny N, Senda J, Saylor KM, Bauermeister JA (2017). The Sexual Health of Transgender Men: A Scoping Review. J Sex Res.

[67] Reisner SL, White JM, Mayer KH, Mimiaga MJ (2014). Sexual risk behaviors and psychosocial health concerns of female-to-male
transgender men screening for STDs at an urban community health center. AIDS Care.

[68] Grant J, Mottet L, Tanis J, Harrison J, Herman J, Keisling M (2011). Injustice at Every Turn: A Report of the National Transgender
Discrimination Survey. National Center for Transgender Equality.

[69] Bockting WO, Miner MH, Swinburne Romine RE, Hamilton A, Coleman E (2013). Stigma, Mental Health, and Resilience in an Online Sample of the US
Transgender Population. Am J Public Health.

[70] Budge SL, Adelson JL, Howard KAS (2013). Anxiety and depression in transgender individuals: the roles of transition
status, loss, social support, and coping. J Consult Clin Psychol.

[71] Hoffman B (2014). An Overview of Depression among Transgender Women. Depress Res Treat.

[72] Igartua KJ, Gill K, Montoro R (2003). Internalized homophobia: a factor in depression, anxiety, and suicide in
the gay and lesbian population. Can J Commun Ment Health.

[73] Nahata L, Quinn GP, Caltabellotta NM, Tishelman AC (2017). Mental Health Concerns and Insurance Denials Among Transgender
Adolescents. LGBT Health.

[74] Nuttbrock L, Bockting W, Rosenblum A, Hwahng S, Mason M, Macri M, Becker J (2014). Gender Abuse and Major Depression Among Transgender Women: A Prospective
Study of Vulnerability and Resilience. Am J Public Health.

[75] Clements-Nolle K, Marx R, Katz M (2006). Attempted Suicide Among Transgender Persons. Journal of Homosexuality.

[76] Mereish EH, O'Cleirigh C, Bradford JB (2014). Interrelationships between LGBT-based victimization, suicide, and substance
use problems in a diverse sample of sexual and gender minorities. Psychol Health Med.

[77] Mustanski B, Liu RT (2013). A longitudinal study of predictors of suicide attempts among lesbian, gay,
bisexual, and transgender youth. Arch Sex Behav.

[78] Reisner SL, Vetters R, Leclerc M, Zaslow S, Wolfrum S, Shumer D, Mimiaga MJ (2015). Mental health of transgender youth in care at an adolescent urban community
health center: a matched retrospective cohort study. J Adolesc Health.

[79] Operario D, Nemoto T (2010). HIV in transgender communities: syndemic dynamics and a need for
multicomponent interventions. J Acquir Immune Defic Syndr.

[80] Baral SD, Poteat T, Strömdahl S, Wirtz AL, Guadamuz TE, Beyrer C (2013). Worldwide burden of HIV in transgender women: a systematic review and
meta-analysis. The Lancet Infectious Diseases.

[81] Melendez RM, Pinto R (2007). 'It's really a hard life': love, gender and HIV risk among male-to-female
transgender persons. Cult Health Sex.

[82] Reisner SL, Pardo ST, Gamarel KE, White Hughto JM, Pardee DJ, Keo-Meier CL (2015). Substance Use to Cope with Stigma in Healthcare Among U.S. Female-to-Male
Trans Masculine Adults. LGBT Health.

[83] Elamin M, Garcia M, Murad M, Erwin P, Montori V (2010). Effect of sex steroid use on cardiovascular risk in transsexual
individuals: a systematic review and meta-analyses. Clin Endocrinol (Oxf).

[84] Wierckx K, Elaut E, Declercq E, Heylens G, De Cuypere G, Taes Y, Kaufman JM, T'Sjoen G (2013). Prevalence of cardiovascular disease and cancer during cross-sex hormone
therapy in a large cohort of trans persons: a case-control study. Eur J Endocrinol.

[85] Feldman J, Brown GR, Deutsch MB, Hembree W, Meyer W, Meyer-Bahlburg HF, Tangpricha V, TʼSjoen G, Safer JD (2016). Priorities for transgender medical and healthcare research. Current Opinion in Endocrinology & Diabetes and Obesity.

[86] Stephenson R, Finneran C (2013). The IPV-GBM scale: a new scale to measure intimate partner violence among
gay and bisexual men. PLoS One.

