# Addendum to: Development and Validation of a Scale to Measure Intimate Partner Violence Among Transgender and Gender Diverse Populations: Protocol for a Linear Three-Phase Study (Project Empower)

**DOI:** 10.2196/28614

**Published:** 2021-05-12

**Authors:** Rob Stephenson, Kieran Todd, Kristi E Gamarel, Erin E Bonar, Sarah Peitzmeier

**Affiliations:** 1 Center for Sexuality and Health Disparities and The School of Nursing University of Michigan Ann Arbor, MI United States; 2 Center for Sexuality and Health Disparities University of Michigan Ann Arbor, MI United States; 3 Center for Sexuality and Health Disparities and The School of Public Health University of Michigan Ann Arbor, MI United States; 4 Addiction Center Department of Psychiatry University of Michigan Ann Arbor, MI United States; 5 The Injury Prevention Center University of Michigan Ann Arbor, MI United States

In “Development and Validation of a Scale to Measure Intimate Partner Violence Among Transgender and Gender Diverse Populations: Protocol for a Linear Three-Phase Study (Project Empower)” (JMIR Res Protoc 2020;9(11):e23819), corrections have been made to the authorship list and to the text of the paper to clarify the methods of the protocol.

Author Erin E Bonar has been added to the authorship list of the revised paper. The revised list of authors and affiliations is as follows:

Rob Stephenson^1*^, MA, MSc, PhD; Kieran Todd^2^, MPH; Kristi E Gamarel^3^, PhD; Erin E Bonar^2,4,5^, PhD; Sarah Peitzmeier^1*^, PhD

^1^Center for Sexuality and Health Disparities and The School of Nursing, University of Michigan, Ann Arbor, MI, United States

^2^Center for Sexuality and Health Disparities, University of Michigan, Ann Arbor, MI, United States

^3^Center for Sexuality and Health Disparities and The School of Public Health, University of Michigan, Ann Arbor, MI, United States

^4^Addiction Center, Department of Psychiatry, University of Michigan, Ann Arbor, MI, United States

^5^The Injury Prevention Center, University of Michigan, Ann Arbor, MI, United States

^*^these authors contributed equally

To clarify the age of the participant group, mentions throughout the paper of participants as “aged over 15 years” have been changed to “aged 15 and above.”

The section “Protection of Human Subjects” has been edited to clarify the risk management plan for participants.

References 40 and 45 in the original manuscript have been removed. 3 new references have been added to the reference list as References 41-43 and are cited in the "Health Outcomes" section. References have been renumbered accordingly. The full revised reference list appears below [1-86].

In the “Health Outcomes” section, the measure of suicidal ideation has been removed. The measure of substance abuse has been changed to the following:

Substance use measures will assess the use and frequency of use of alcohol and other drugs in the past 3 months and are based on prior work [41-44].

The correction will appear in the online version of the paper on the JMIR Publications website on May 12, 2021, together with the publication of this correction notice. Because this was made after submission to PubMed, PubMed Central, and other full-text repositories, the corrected article has also been resubmitted to those repositories.

## References

[1] Peitzmeier SM, Malik M, Kattari SK, Marrow E, Stephenson R, Agénor M, Reisner SL (2020). Intimate Partner Violence in Transgender Populations: Systematic Review and Meta-analysis of Prevalence and Correlates. Am J Public Health.

[2] James S, Herman J, Rankin S, Keisling M, Mottet L, Anafi M (2016). The Report of the 2015 U.S. Transgender Survey.

[3] Black M, Basile K, Breiding M, Smith S, Walters M, Merrick M, Chen J, Stevens M (2011). The National Intimate Partner and Sexual Violence Survey (NISVS): 2010 Summary Report.

[4] Reisner SL, White Hughto JM, Gamarel KE, Keuroghlian AS, Mizock L, Pachankis JE (2016). Discriminatory experiences associated with posttraumatic stress disorder symptoms among transgender adults. J Couns Psychol.

[5] White Hughto JM, Pachankis JE, Willie TC, Reisner SL (2017). Victimization and depressive symptomology in transgender adults: The mediating role of avoidant coping. J Couns Psychol.

[6] Goldenberg T, Jadwin-Cakmak L, Harper GW (2018). Intimate Partner Violence Among Transgender Youth: Associations with Intrapersonal and Structural Factors. Violence Gend.

[7] Logie CH, Wang Y, Lacombe-Duncan A, Jones N, Ahmed U, Levermore K, Neil A, Ellis T, Bryan N, Marshall A, Newman PA (2017). Factors associated with sex work involvement among transgender women in Jamaica: a cross-sectional study. Journal of the International AIDS Society.

[8] Straus MA, Hamby SL, Boney-McCoy S, Sugarman DB (2016). The Revised Conflict Tactics Scales (CTS2). Journal of Family Issues.

[9] Munson M, Cook-Daniels L (2003). Transgender/SOFFA: Domestic Violence/Sexual Assault Resource Sheet.

[10] Guadalupe-Diaz XL, Anthony AK (2016). Discrediting Identity Work: Understandings of Intimate Partner Violence by Transgender Survivors. Deviant Behavior.

[11] Woulfe JM, Goodman LA (2018). Identity Abuse as a Tactic of Violence in LGBTQ Communities: Initial Validation of the Identity Abuse Measure. J Interpers Violence.

[12] Peitzmeier SM, Hughto JM, Potter J, Deutsch MB, Reisner SL (2019). Development of a Novel Tool to Assess Intimate Partner Violence Against Transgender Individuals. J Interpers Violence.

[13] Peitzmeier S, Reisner S, Cooney E, Humes E, Wirtz A (2019). Validation of a scale to measure transgender-specific psychological intimate partner violence.

[14] Dyar C, Messinger AM, Newcomb ME, Byck GR, Dunlap P, Whitton SW (2019). Development and Initial Validation of Three Culturally Sensitive Measures of Intimate Partner Violence for Sexual and Gender Minority Populations. J Interpers Violence.

[15] Hendricks M, Testa R (2012). A conceptual framework for clinical work with transgender and gender nonconforming clients: An adaptation of the Minority Stress Model. Professional Psychology: Research and Practice.

[16] Sevelius JM (2013). Gender Affirmation: A Framework for Conceptualizing Risk Behavior among Transgender Women of Color. Sex Roles.

[17] Glynn TR, Gamarel KE, Kahler CW, Iwamoto M, Operario D, Nemoto T (2016). The role of gender affirmation in psychological well-being among transgender women. Psychol Sex Orientat Gend Divers.

[18] Gamarel KE, Reisner SL, Laurenceau J, Nemoto T, Operario D (2014). Gender minority stress, mental health, and relationship quality: a dyadic investigation of transgender women and their cisgender male partners. J Fam Psychol.

[19] Reisner SL, Gamarel KE, Nemoto T, Operario D (2014). Dyadic effects of gender minority stressors in substance use behaviors among transgender women and their non-transgender male partners. Psychol Sex Orientat Gend Divers.

[20] Patton MQ (1990). Qualitative Research & Evaluation Methods.

[21] Dworkin S (2012). Sample size policy for qualitative studies using in-depth interviews. Arch Sex Behav.

[22] Beatty P, Willis G (2007). Research Synthesis: The Practice of Cognitive Interviewing. Public Opinion Quarterly.

[23] Graham-Kevan N, Archer J (2003). Intimate terrorism and common couple violence. A test of Johnson's predictions in four British samples. J Interpers Violence.

[24] Testa R, Habarth J, Peta J, Balsam K, Bockting W (2015). Development of the Gender Minority Stress and Resilience Measure. Psychology of Sexual Orientation and Gender Diversity.

[25] Scheim AI, Bauer GR (2019). The Intersectional Discrimination Index: Development and validation of measures of self-reported enacted and anticipated discrimination for intercategorical analysis. Soc Sci Med.

[26] Clark R, Coleman AP, Novak JD (2004). Brief report: Initial psychometric properties of the everyday discrimination scale in black adolescents. J Adolesc.

[27] Forman TA, Williams DR, Jackson JS, Gardner C (1997). Race, place, and discrimination. Perspectives on Social Problems.

[28] Essed P (1991). Analyzing Accounts of Racism. Understanding Everyday Racism: An Interdisciplinary Theory.

[29] Brondolo E, Kelly KP, Coakley V, Gordon T, Thompson S, Levy E, Cassells A, Tobin JN, Sweeney M, Contrada RJ (2005). The Perceived Ethnic Discrimination Questionnaire: Development and Preliminary Validation of a Community Version1. J Appl Social Pyschol.

[30] Contrada RJ, Ashmore RD, Gary ML, Coups E, Egeth JD, Sewell A, Ewell K, Goyal TM, Chasse V (2001). Measures of Ethnicity-Related Stress: Psychometric Properties, Ethnic Group Differences, and Associations With Well-Being1. J Appl Social Pyschol.

[31] Swim J, Cohen L, Hyers L (1998). Experiencing everyday prejudice and discrimination. Prejudice: The Target's Perspective.

[32] Piggott M (2004). Double jeopardy: Lesbians and the legacy of multiple stigmatized identities. Swinburne University.

[33] Szymanski DM, Dunn TL, Ikizler AS (2014). Multiple minority stressors and psychological distress among sexual minority women: The roles of rumination and maladaptive coping. Psychology of Sexual Orientation and Gender Diversity.

[34] Sevelius J, Chakravarty D, Neilands TB, Keatley J, Shade SB, Johnson MO, Rebchook G, HRSA SPNS Transgender Women of Color Study Group (2019). Evidence for the Model of Gender Affirmation: The Role of Gender Affirmation and Healthcare Empowerment in Viral Suppression Among Transgender Women of Color Living with HIV. AIDS Behav.

[35] Bockting WO, Miner MH, Swinburne Romine RE, Hamilton A, Coleman E (2013). Stigma, Mental Health, and Resilience in an Online Sample of the US Transgender Population. Am J Public Health.

[36] Spanier GB (1976). Measuring Dyadic Adjustment: New Scales for Assessing the Quality of Marriage and Similar Dyads. Journal of Marriage and the Family.

[37] Tzeng OC (1994). Family Relations. Measurement of Love and Intimate Relations: Theories, Scales, and Applications for Love Development, Maintenance, and Dissolution.

[38] Sherman S, Gielen A, McDonnell K (2000). Brief Report: Power and Attitudes in Relationships (PAIR) Among a Sample of Low-Income, African-American Women: Implications for HIV/AIDS Prevention. Sex Roles.

[39] Reisner S, Deutsch M, Cavanaugh T, Pardee D, White HJ, Peitzmeier S, McLean S, Mimiaga M, Panther L, Potter J (2016). Best practices for obtaining a sexual health history with trans masculine individuals: Lessons learned from self-administered surveys and provider-collected clinical interview data. Proceedings of the 24th World Professional Association for Transgender Health Biennial Symposium: Growing Empowerment, Expertise, Evidence; WPATH'16.

[40] Saunders J, Aasland O, Babor T, de la Fuente JR, Grant M (1993). Development of the Alcohol Use Disorders Identification Test (AUDIT): WHO Collaborative Project on Early Detection of Persons with Harmful Alcohol Consumption--II. Addiction.

[41] NIDA Alcohol, Smoking, and Substance Involvement Screening Test: NM-ASSIST.

[42] WHO ASSIST Working Group (2002). The Alcohol, Smoking and Substance Involvement Screening Test (ASSIST): development, reliability and feasibility. Addiction.

[43] Grant BF, Chu A, Sigman R, Amsbary M, Kali J, Sugawara Y, Jiao R, Ren W, Goldstein R (2003). National Epidemiologic Survey on Alcohol and Related Conditions-III (NESARC- III): Source and Accuracy Statement.

[44] Derogatis LR, Melisaratos N (2009). The Brief Symptom Inventory: an introductory report. Psychol. Med.

[45] Ouimette P, Wade M, Prins A, Schohn M (2008). Identifying PTSD in primary care: comparison of the Primary Care-PTSD screen (PC-PTSD) and the General Health Questionnaire-12 (GHQ). J Anxiety Disord.

[46] Primary Care PTSD Screen for DSM-5 (PC-PTSD-5). U.S. Department of Veterans Affairs.

[47] Cella D, Riley W, Stone A, Rothrock N, Reeve B, Yount S, Amtmann D, Bode R, Buysse D, Choi S, Cook K, Devellis R, DeWalt D, Fries JF, Gershon R, Hahn EA, Lai J, Pilkonis P, Revicki D, Rose M, Weinfurt K, Hays R, PROMIS Cooperative Group (2010). The Patient-Reported Outcomes Measurement Information System (PROMIS) developed and tested its first wave of adult self-reported health outcome item banks: 2005-2008. J Clin Epidemiol.

[48] Hegarty K, Bush R, Sheehan M (2005). The Composite Abuse Scale: Further Development and Assessment of Reliability and Validity of a Multidimensional Partner Abuse Measure in Clinical Settings. Violence.

[49] Tabachnick B, Fidell L (2007). Using multivariate statistics.

[50] Cudeck R, Tinsley H, Brown S (2000). Exploratory factor analysis. Handbook of Applied Multivariate Statistics and Mathematical Modeling.

[51] Hinkin TR (2016). A Brief Tutorial on the Development of Measures for Use in Survey Questionnaires. Organizational Research Methods.

[52] DeVellis R (2017). Scale development: Theory and applications.

[53] Yu CY, Muthen BO (2002). Evaluation of model fit indices for latent variable models with categorical and continuous outcomes.

[54] Schumacker R, Lomax R (2004). A Beginner's Guide to Structural Equation Modeling.

[55] Snijders T, Bosker R (1999). Multilevel analysis: An introduction to basic and applied multilevel analysis. Multilevel Analysis.

[56] Durwood L, McLaughlin KA, Olson KR (2017). Mental Health and Self-Worth in Socially Transitioned Transgender Youth. J Am Acad Child Adolesc Psychiatry.

[57] Valentine SE, Peitzmeier SM, King DS, O'Cleirigh C, Marquez SM, Presley C, Potter J (2017). Disparities in Exposure to Intimate Partner Violence Among Transgender/Gender Nonconforming and Sexual Minority Primary Care Patients. LGBT Health.

[58] Seelman KL, Colón-Diaz MJ, LeCroix RH, Xavier-Brier M, Kattari L (2017). Transgender Noninclusive Healthcare and Delaying Care Because of Fear: Connections to General Health and Mental Health Among Transgender Adults. Transgend Health.

[59] Bockting W, Coleman E, Deutsch MB, Guillamon A, Meyer I, Meyer W, Reisner S, Sevelius J, Ettner R (2016). Adult development and quality of life of transgender and gender nonconforming people. Current Opinion in Endocrinology & Diabetes and Obesity.

[60] (2017). HIV and Transgender People. Centers for Disease Control and Prevention.

[61] Gamarel KE, Reisner SL, Laurenceau J, Nemoto T, Operario D (2014). Gender minority stress, mental health, and relationship quality: a dyadic investigation of transgender women and their cisgender male partners. J Fam Psychol.

[62] Meyer IH, Brown TN, Herman JL, Reisner SL, Bockting WO (2017). Demographic Characteristics and Health Status of Transgender Adults in Select US Regions: Behavioral Risk Factor Surveillance System, 2014. Am J Public Health.

[63] Reisner SL, Gamarel KE, Dunham E, Hopwood R, Hwahng S (2013). Female-to-male transmasculine adult health: a mixed-methods community-based needs assessment. J Am Psychiatr Nurses Assoc.

[64] Reisner SL, Greytak EA, Parsons JT, Ybarra ML (2015). Gender minority social stress in adolescence: disparities in adolescent bullying and substance use by gender identity. J Sex Res.

[65] Reisner SL, Katz-Wise SL, Gordon AR, Corliss HL, Austin SB (2016). Social Epidemiology of Depression and Anxiety by Gender Identity. J Adolesc Health.

[66] Stephenson R, Riley E, Rogers E, Suarez N, Metheny N, Senda J, Saylor KM, Bauermeister JA (2017). The Sexual Health of Transgender Men: A Scoping Review. J Sex Res.

[67] Reisner SL, White JM, Mayer KH, Mimiaga MJ (2014). Sexual risk behaviors and psychosocial health concerns of female-to-male transgender men screening for STDs at an urban community health center. AIDS Care.

[68] Grant J, Mottet L, Tanis J, Harrison J, Herman J, Keisling M (2011). Injustice at Every Turn: A Report of the National Transgender Discrimination Survey. National Center for Transgender Equality.

[69] Bockting WO, Miner MH, Swinburne Romine RE, Hamilton A, Coleman E (2013). Stigma, Mental Health, and Resilience in an Online Sample of the US Transgender Population. Am J Public Health.

[70] Budge SL, Adelson JL, Howard KAS (2013). Anxiety and depression in transgender individuals: the roles of transition status, loss, social support, and coping. J Consult Clin Psychol.

[71] Hoffman B (2014). An Overview of Depression among Transgender Women. Depress Res Treat.

[72] Igartua KJ, Gill K, Montoro R (2003). Internalized homophobia: a factor in depression, anxiety, and suicide in the gay and lesbian population. Can J Commun Ment Health.

[73] Nahata L, Quinn GP, Caltabellotta NM, Tishelman AC (2017). Mental Health Concerns and Insurance Denials Among Transgender Adolescents. LGBT Health.

[74] Nuttbrock L, Bockting W, Rosenblum A, Hwahng S, Mason M, Macri M, Becker J (2014). Gender Abuse and Major Depression Among Transgender Women: A Prospective Study of Vulnerability and Resilience. Am J Public Health.

[75] Clements-Nolle K, Marx R, Katz M (2006). Attempted Suicide Among Transgender Persons. Journal of Homosexuality.

[76] Mereish EH, O'Cleirigh C, Bradford JB (2014). Interrelationships between LGBT-based victimization, suicide, and substance use problems in a diverse sample of sexual and gender minorities. Psychol Health Med.

[77] Mustanski B, Liu RT (2013). A longitudinal study of predictors of suicide attempts among lesbian, gay, bisexual, and transgender youth. Arch Sex Behav.

[78] Reisner SL, Vetters R, Leclerc M, Zaslow S, Wolfrum S, Shumer D, Mimiaga MJ (2015). Mental health of transgender youth in care at an adolescent urban community health center: a matched retrospective cohort study. J Adolesc Health.

[79] Operario D, Nemoto T (2010). HIV in transgender communities: syndemic dynamics and a need for multicomponent interventions. J Acquir Immune Defic Syndr.

[80] Baral SD, Poteat T, Strömdahl S, Wirtz AL, Guadamuz TE, Beyrer C (2013). Worldwide burden of HIV in transgender women: a systematic review and meta-analysis. The Lancet Infectious Diseases.

[81] Melendez RM, Pinto R (2007). 'It's really a hard life': love, gender and HIV risk among male-to-female transgender persons. Cult Health Sex.

[82] Reisner SL, Pardo ST, Gamarel KE, White Hughto JM, Pardee DJ, Keo-Meier CL (2015). Substance Use to Cope with Stigma in Healthcare Among U.S. Female-to-Male Trans Masculine Adults. LGBT Health.

[83] Elamin M, Garcia M, Murad M, Erwin P, Montori V (2010). Effect of sex steroid use on cardiovascular risk in transsexual individuals: a systematic review and meta-analyses. Clin Endocrinol (Oxf).

[84] Wierckx K, Elaut E, Declercq E, Heylens G, De Cuypere G, Taes Y, Kaufman JM, T'Sjoen G (2013). Prevalence of cardiovascular disease and cancer during cross-sex hormone therapy in a large cohort of trans persons: a case-control study. Eur J Endocrinol.

[85] Feldman J, Brown GR, Deutsch MB, Hembree W, Meyer W, Meyer-Bahlburg HF, Tangpricha V, TʼSjoen G, Safer JD (2016). Priorities for transgender medical and healthcare research. Current Opinion in Endocrinology & Diabetes and Obesity.

[86] Stephenson R, Finneran C (2013). The IPV-GBM scale: a new scale to measure intimate partner violence among gay and bisexual men. PLoS One.

